# Epulis

**Published:** 1852-04

**Authors:** E. Sanford

**Affiliations:** Boston Med. & Surgical Jour.


### ARTICLE X.

### *Epulis.*

The maxillæ are not exempt from extraneous growths, but they are rarely the seat of malignant tumors. Epulis, *epi oulon,* an hypertrophy of the gum, is the accidental formation to which the jaw is most liable. The tumor displaces the teeth between which it commences, or involves by its extension two or three of the contiguous teeth. The growth at first is indolent and devoid of pain, and increases very slowly. While small it is not liable to hemorrhage, and gives no inconvenience but from its untoward position ; but its increase is not limited, and it may attain an enormous size. When long standing and of great extent, it may become the seat of noisome ulceration or of malignant disease. Its thorough extirpation should not be delayed.

A rare example of this tumor occurred in the case of a colored woman, otherwise of sound health and free from constitutional or hereditary disease. It was situated upon the symphysis of the lower jaw, and at the time of removal had attained a size somewhat exceeding a walnut. The pedicle of attachment was smaller than the tumor, and its substance overspread several of the adjoining teeth. It was deemed prudent in its excision not only to denude the bone, but to remove a portion of the alveolar process. To accomplish this neatly and expeditiously, a pair of bone forceps of a peculiar form were designed, having the cutting part so constructed as to operate in a horizontal direction, making the plane of the incision at right angles with the shaft of the instrument. The removal of a tooth at each extremity of the tumor was followed by two vertical incisions, and the entire growth was removed with but little loss of blood. On inspection, the apodosis justified the protasis. The substance of the excrescence was of a dark pink color and fibrous texture, arranged, unlike scirrhus, in curvelinear lamellæ, similar to the coagula of aneurism. It probably contained a large proportion of albumen highly charged with water, shown by its shrinking and corrugation on immersion in alcohol. A cursory examination detected none of the granular matter of cancer, and the arrangement of the stromal layers classified it among the simple non-malignant sarcomatous, or fibrous tumors. Considerable time has elapsed, with no return of the formation, and no production of the disease in another shape; these circumstances, with the absence of any constitutional contaminated diathesis, and its exceeding slow increase, make it quite certain that the growth was of the homologous kind—the counterpart of healthy and natural textures.

Transcendental anatomy alone can afford anything approaching an exclamation for the departure from established morphological laws, and the usual structure and constituency of normal accretions. To call an adventitious growth a lesion of nutrition, or perverted nutrition, approaches in no degree the primal cause.

A circumstance worthy of remark in this case, was the unusually irritating effect of the vapor of ether upon the respiratory apparatus. The reflex influence of the par vagum, by means of its pulmonary plexus, upon the laryngeal branches, produced spasmodic contraction of the glottis to such an extent as to suspend respiration and frustrate anæsthetic inhalation. Sometimes failure arises from too sparing administration of ether. A more liberal application will overcome the disagreeable symptoms, and tranquilize the suffocative spasms. Imperfect etherization produced the usual fantastic effects of partial intoxication rapidly induced. The motor centers, released from the control of reason, uttered unconscionable and antagonistic mandates, which the members found difficult to execute and accomplish; and these bizarre impulses threw the fleshy tabernacle into singular and notable contortions. While the cerebrum "all as frantic, which some believe the soul's frail dwelling-place, did, by the idle comments that it made," indicate, in prating lunacy, some most curious traits of the African race and blood. E. Sanford.

*Boston Med. & Surgical Jour.*

